# Salience Matters: Filler groups on the ascent of human scale impact ratings for target groups

**DOI:** 10.1371/journal.pone.0293398

**Published:** 2023-11-10

**Authors:** Devin L. Johnson, Sukhvinder S. Obhi

**Affiliations:** Department of Psychology, Neuroscience, and Behaviour, McMaster University, Hamilton, Canada; Pontificia Universidad Catolica de Chile (PUC) / Universidad de Valladolid (UVa), SPAIN

## Abstract

Researchers using the ascent of human scale (AOH) to study dehumanization typically include filler groups in addition to the main comparator groups, to hide the true intent of the study. However, there is little work examining the impact of filler group choice on dehumanization ratings between groups of interest. Across two studies (including one pre-registered study) we manipulated the salience of a target out-group (i.e., the extent to which the group stood out) by embedding it within lists of other groups. By comparing AOH ratings across three conditions in which the target out-group was either high salience, medium salience, or low salience, we were able to determine the effects of target out-group salience on dehumanization. In study 1, we included participants’ in-group (Canadian) in the list, and in study 2, we did not include participants in-group in the list. Results from study 1 showed that group salience had no impact on AOH ratings for the out-group when the participant in-group was included in the list. However, in study 2, when participant in-group was removed from the list, ratings for the out-group in the high salience condition were significantly lower than both the medium and low salience conditions. Implications for both theoretical and methodological issues in investigations using the AOH scale are discussed.

## Introduction

Within the psychological literature, dehumanization can be defined as the act of seeing others as less then human and denying them human faculties [1, 2] Sadly, dehumanization persists across societies and is directed towards many social groups [3]. Early work on the psychology of dehumanization was interested in blatant forms of the phenomena such as directly describing others as animals or vermin [4] as such tactics were applied in the atrocities of World War II. However, as dehumanization research moved into laboratory settings scholars focused on using indirect measures, with the general assumption being that study participants would not willingly express the extent to which they view other groups or individuals as less than human. For example, variations on the implicit association task have been used to measure the extent to which people associate out-groups with animals [5]. Other measures focus on the degree to which participants ascribe mental faculties to out-groups such as the capacity make purposeful action, or feel emotion [6].

A popular measure was derived from work in infrahumanization theory which posits that individuals are more willing to ascribe uniquely human emotions to their in-group in relation to an out-group [7]. Here participants rate the extent to which they feel their in-group as well as out-groups of interest are capable of feeling emotions argued to be unique to the human experience. Building off this [1] proposed the dual model theory of dehumanization which argues that people can be animalistically as well as mechanistically dehumanized, likened more to either animals or machines. For this measure, participants rate the how much individuals or groups can embody specific traits argued to be unique to humans exclusively, or traits that both humans and animals can possess. When participants rate others as less capable of experiencing uniquely human emotions or embodying uniquely human traits, it reflects a denial of a fundamental aspect of humanity [2, 3].

**Fig 1:**
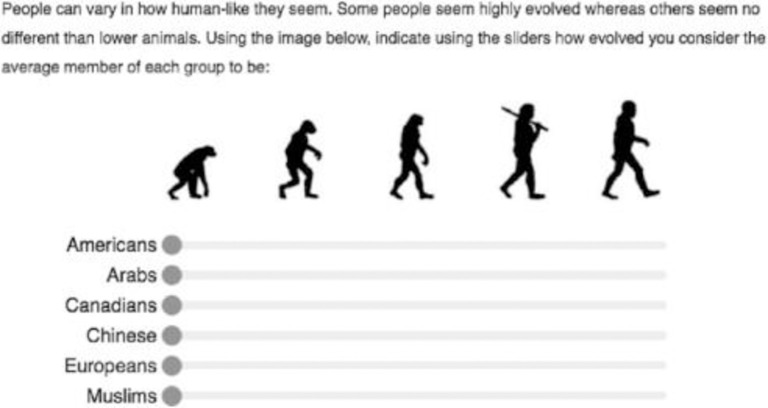
Ascent of human scale as introduced in Kteily et al, (2015).

Recently work within social psychology has re-focused efforts to measure blatant forms of dehumanization. One such measure is the ascent of (Hu)man scale (AOH), formerly known as the “Ascent of Man Scale” introduced by [8]. In this scale participants are presented with the classic Ascent of Man image representing 5 pictures that show a lower order primate on the left progressing to that of an upright human on the right. Participants rate various social groups on a slider scale from 0 (indicating least evolved) to 100 (most evolved) which corresponds to the Ascent of Man image. See Fig 1 for a depiction of the AOH scale. Results from various studies using the AOH scale show that blatant forms of dehumanization are more prevalent than previously assumed with results showing that participants are more than willing to rate social groups as less evolved than others, especially their in-group [8, 9]. Studies have demonstrated that blatant dehumanization of this type is not something limited to racial or ethnic groups but can be directed towards a host of out groups such as people with obesity [10], individuals who are short in stature [11], people on drugs such as heroin [12], and persons who are homeless [13] to name a few.

Studies have also shown the utility of ascent dehumanization not only as a dependent variable, measuring how much out-groups are dehumanized, but also as a useful independent variable, especially when related to predicting attitudes and behaviors considered crucial within inter-group relations and conflict. One consistent finding has been that ascent dehumanization is related to social dominance orientation, the preference for group-based hierarchy [14]. The more individuals prefer group-based hierarchies and are in favor of their maintenance through overt violence of institutional norms, the greater they tend to dehumanize out-groups via the AOH scale [8, 15, 16]. Ascent dehumanization has also been found to be predictive of the support for the torture of terror suspects by Americans [8], the acceptance of out-group collateral damage in open conflicts [16], and the extent to which teachers will deny support towards students they dehumanize [17]. Additionally, AOH scores are predictive of attitudes toward outgroups, even when accounting for negative attitudes such as prejudice, as measured by the feeling thermometer [8]. As such the AOH scale has proven useful in allowing researchers to better understand blatant dehumanization, specifically how persistent it is as well as how damaging it can be to those on the receiving end of it.

While [8] report rigorous work conducted in order to validate the scale, recent research has begun to investigate potential issues pertaining to how the scale is implemented in experiments. In a large sample, pre-registered study [18] examined multiple aspects of the AOH scale and variations to how it can be administered. Specifically, the researchers sought to determine if there would be differences in results when individual groups were presented to participants one at a time or all together, the latter being the more common approach used in studies with the AOH scale. Researchers also sought to determine if a potential anchoring effect was present depending on the initial start point for the slider, traditionally placed at the far left of the scale, and if presenting the scale before or after other measures impacted ratings [18]. Results from their study indicate these issues have no impact on overall ratings participants gave, further adding credence to the robustness of the AOH scale in measuring blatant dehumanization [18].

In previous research, authors have included separate validation studies of the scale within their larger research projects. For example, [19] sought to examine how dehumanization scores via the AOH scale would be different when adopting a forced choice task as opposed to a slider scale. In this experiment participants in the forced choice condition had to select one of the five discrete pictures on the Ascent of Man image in order to indicate where they believed the out-group (in that study–Mexican immigrants) to be in terms of evolvedness. This was in contrast to the usual slider scale condition where a participant could place their ranking in between images to varying degrees [19]. Results from that study show lower rates of dehumanization in the forced choice condition compared to the traditional slider scale condition [19]. Research has shown that reaction times and click ratios vary between forced choice and slider scale options on surveys [20]. It would appear that requiring participants to make a distinct choice as to where groups are in the hierarchy of images on the ascent scale inhibits the willingness to dehumanize.

Typically, the AOH scale is presented with an instruction prompt that begins with the claim that some groups are more evolved than others. As such research has also sought to determine the extent to which the scale’s instructions have an impact on ratings. In their initial article presenting the scale [8] conducted a validation study, comparing AOH ratings when the instructions were present or omitted. Results indicate that the removal of the text instructions had no impact on scale results. Other studies have sought to address how mentions of evolution in the instructions affect ratings. In two separate studies [21, 22] conducted validation studies where created conditions in which participants took the AOH scale but without language specifying evolution in the instructions. Results from both studies suggest that removing evolutionary charged language does not impact how participants rate groups on the scale when compared to its traditional version [21, 22].

It is also common for many studies using the AOH scale to include additional groups to be rated, with the intention of concealing the comparisons of interest from study participants. These additional groups meant to hide study intentions have been dubbed “Filler Groups” in the literature [17].

Some studies explicitly mention the use of filler groups in order to hide the true intention of the study to participants [10, 12]. Others mention their use of filler groups but make no explicit mention of which ones they’ve used [23]. However, not every paper that uses the AOH scale applies this method, with some articles simply presenting the groups of interest and no addition of filler groups [24]. As such, it would appear that the use of filler groups is not a requirement for using the AOH scale. In fact, [8] first used many groups on the scale in a catch all manner, to see which demographic groups would be dehumanized.

Curiously, the impact of filler groups, and more specifically filler group choice has been one aspect of the scale not investigated to our knowledge. One area of concern may lie in salience, denoting the degree to which an object or attribute exhibits conspicuousness, particularly within the context of social and ethnic categorizations. Prior research has demonstrated that social cues pertaining to individuals, such as their skin color [25] or the ethnic provenance implied by their name [26], exert a discernible influence on the manner in which others engage with these individuals. This phenomenon is particularly pronounced when such cues deviate from the prevailing social categories that typify predominant groups within a given locale [25–27].

This is a potential concern as traditionally, the AOH scale is administered in a way in which groups are presented all at once to be rated [18]. Research in psychology has shown how salience impacts attention such that the more something stands out, the greater attention it receives [28, 29]. Moreover, research on attention has shown that bias in rating scales may arise through sequencing effects related to the manner in which specific attributes of preceding stimuli contrast with novel stimuli in the context of rating tasks [30]. Within inter-group relations, research has demonstrated that demographic salience such as race and gender can impact how others are perceived and treated. Salience, or the degree to which a person stands out from others due to some aspect of their physical appearance, can have adverse effects with research showing the more an individual stands out the more they can be subjected to prejudice in social settings such as hiring [27]. This is of direct concern to researchers using the AOH scale as much work has been done to determine blatant dehumanization of marginalized groups such as Muslims [9] and Roma [17]. Within studies using the AOH scale, it is customary to incorporate multiple social groups defined by distinct identity attributes. In such instances, the prominence of specific groups in relation to others, whether in terms of social status or other relevant factors such as race or religion for example, has the potential to invoke social comparisons among participants. These comparisons may lead participants to evaluate the groups in a relative context, comparing them to one another, rather than assessing each group in isolation.

Ultimately the use of filler groups, and which ones to include, appear to depend on the decisions of individual research teams. Ideally, a standardized process of when or when not to use filler groups, as well as how similar or dissimilar they need to be from the target groups of interest, would greatly improve future studies interested in using the scale.

### Purpose of this study

Given the lack of a standardized process for use of filler groups in studies using the AOH scale, and the gap in the literature assessing how filler group choice can affect results, this study sought to investigate filler group selection and its impact on AOH ratings. To do this we focused on social category salience, extent to which a group stands out in relation to other social groups, as a first step in examining filler group choice.

To accomplish this, we sought to investigate ascent dehumanization of Arabs, a group marginalized within a Western context, and a group that has been subjected to blatantl dehumanization by study participants in this region [16, 31]. In two studies (the first pre-registered), we presented participants with the AOH scale in which the group “Arabs” was made to stand out in comparison to other groups that vary in their level of margninalization within a Canadian context. This was done by varying the presence of other low- status groups who have also been subject to dehumanization. This approach allowed us to create three salience conditions in which the target group “Arabs” stood out a lot (high salience), did not stand out (low salience), or was somewhere in between these extremes (medium salience).

## Materials & methods

### Study 1

#### Pre-registration

Sample size justification as well analysis plan was pre-registered with a link to our plan here: https://aspredicted.org/147_QZL.

#### Sample size

A power analysis conducted using GPower revealed a minimum of 161 participants required to detect a medium effect size (F = .25) for a one-way ANOVA model. As such we aimed for small to medium oversampling.

#### Participants

217 undergraduate McMaster University students were recruited for this study during September of 2022. We included an attention check during the administration of the 7th version of the social dominance orientation scale (SDO) [32]. Those who either failed the attention check or did not confirm their consent in using their data post-debrief were excluded from the final data set. This left us with a total of 180 participants (Mean age = 19.0, SD = 3.3) 77% women.

#### Procedure

Participants were sent a link to an online study where they first read a letter of information and signed a consent form. We had no access to information that could identify individual participants during or after data collection.

Participants were randomly sent to one of the three salience groups; High Salience (HS), Medium Salience (MS), and Low Salience (LS). They were presented with the AOH scale with seven groups presented all together, in random order. In the high salience (HS) condition, our target out-group, Arabs was meant to stand out amongst the other groups. In the medium salience (MS) condition we included similar groups known to experience prejudice and discrimination, balancing for a 50 / 50 split in terms of similarity/ dissimilarity. To accomplish this we focused on including various immigrant groups known to experience prejudice and discrimination in Western countries [33]. Finally in our Low Salience (LS) group, all groups presented were those known to experience prejudice and discrimination so Arabs would not stand out. After providing their ratings on the AOH scale, participants were then asked if any of the groups presented stood out to them amongst the other groups and to specify which ones. They then took the social dominance orientation scale [32]. followed by a series of demographic questions including age, gender, and political orientation on a scale from very liberal to very conservative. See Table 1 for a breakdown of the groups used per salience condition.

**Table 1:** Salience conditions with accompanying ‘filler groups’. All groups presented in random order.

High Salience	Medium Salience	Low Salience
• Germans	• Australians	• Indigenous
• Swedish	• Somalians	• Somalians
• Australians	• Arabs	• Afghans
• Canadians	• New Zealanders	• Syrians
• Arabs	• Afghans	• Arabs
• British	• Canadians	• Canadians
• New Zealanders	• British	• Chinese

### Design and analysis

Our primary goal for this study was to examine the difference in relative dehumanization, the difference between how participants rated their in-group, Canadians, and Arabs across the three salience conditions. In addition, we were also interested in the difference in raw AOH scores for Arabs across the three salience conditions. Studies using the AOH scale typically employ both measures as dependent variables as well as predictor variables [8]. We conducted two one-way analysis of variance (ANOVA) models, one for each research question. All data was analyzed using R Version 4.01.

## Results

### Relative dehumanization

We first examined group salience on relative dehumanization, calculated by computing the difference between AOH ratings for the in-group (Canadians) minus the out-group (Arabs). Results from a one-way ANOVA revealed no effect of salience condition on *relative* dehumanization F(2, 175) = 1.60, p = 0.204; Eta2 = 0.02, 95% CI [0.00, 1.00]. Examination of our model revealed violations in homogeneity of variance via Bartlett’s test (p < .001) as well as violations with regard to normality of model residuals by way of Shapiro’s test (p < .001). To address these violated assumptions in our main model we conducted a Kruskal Wallis rank sum test on relative dehumanization across salience condition. Results revealed a non-significant effect of salience condition on relative dehumanization H(2) = 3.39, p = .18, in line with our parametric analysis.

### Out-group ratings across salience conditions

A one-way ANOVA on ratings for Arabs on the AOH scale also revealed no significant effect of salience condition F(2, 176) = 1.71, p = 0.183; Eta2 = 0.02, 95% CI [0.00, 1.00].

**Fig 2:**
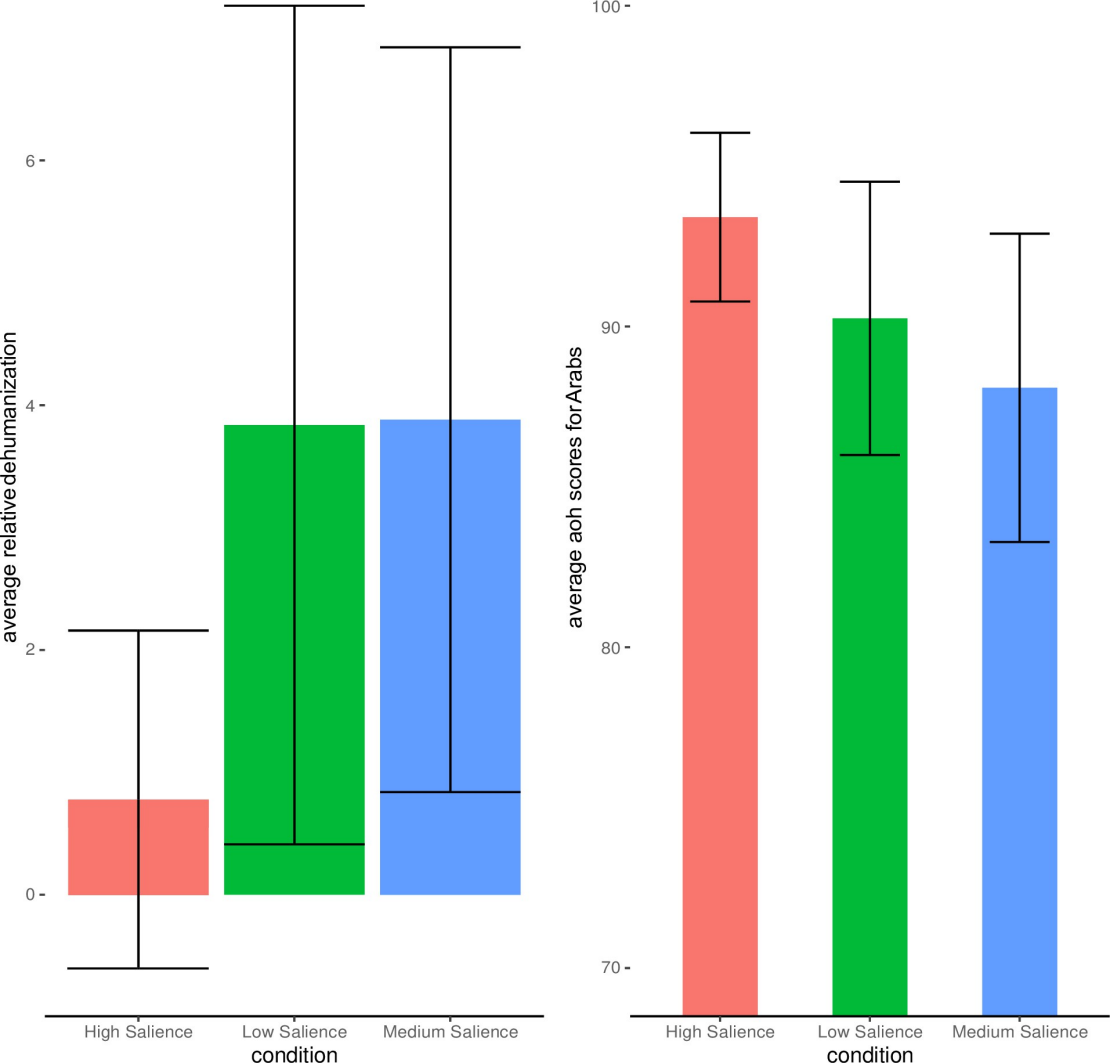
Study 1- Relative Dehumanization between Canadians and Arabs (Left) Ascent of Human ratings for Arabs (Right). Error Bars represent 95% Confidence Intervals.

As in our model for relative dehumanization we detected violations for homogeneity of variance (p < .001), as well as normality (p < .001) by way of Bartlett’s and Shapiro’s test respectively. We again conducted Kruskal Wallis rank sum test to address these violations. Results revealed no significant effect of salience condition on AOH scores for Arabs H(2) = 1.04, p = .59. See Fig 2 for a visualization of these results.

### Exploratory analysis

In the interest of measuring the extent of dehumanization towards Arabs in our study we also conducted a 3 x 2 Mixed ANOVA with salience condition as a between subjects factor, and AOH ratings for Canadians and Arabs as a within subject factor, classified as ‘group’. Results revealed a main effect of group F(1, 175) = 11.56, p <. 001, ETA2 = .010 with Arabs rated significantly lower than Canadians, indicating dehumanization towards Arabs. There was no main effect of salience condition (p = .48), nor an interaction effect between salience condition and ascent group (p = .24).

**Fig 3:**
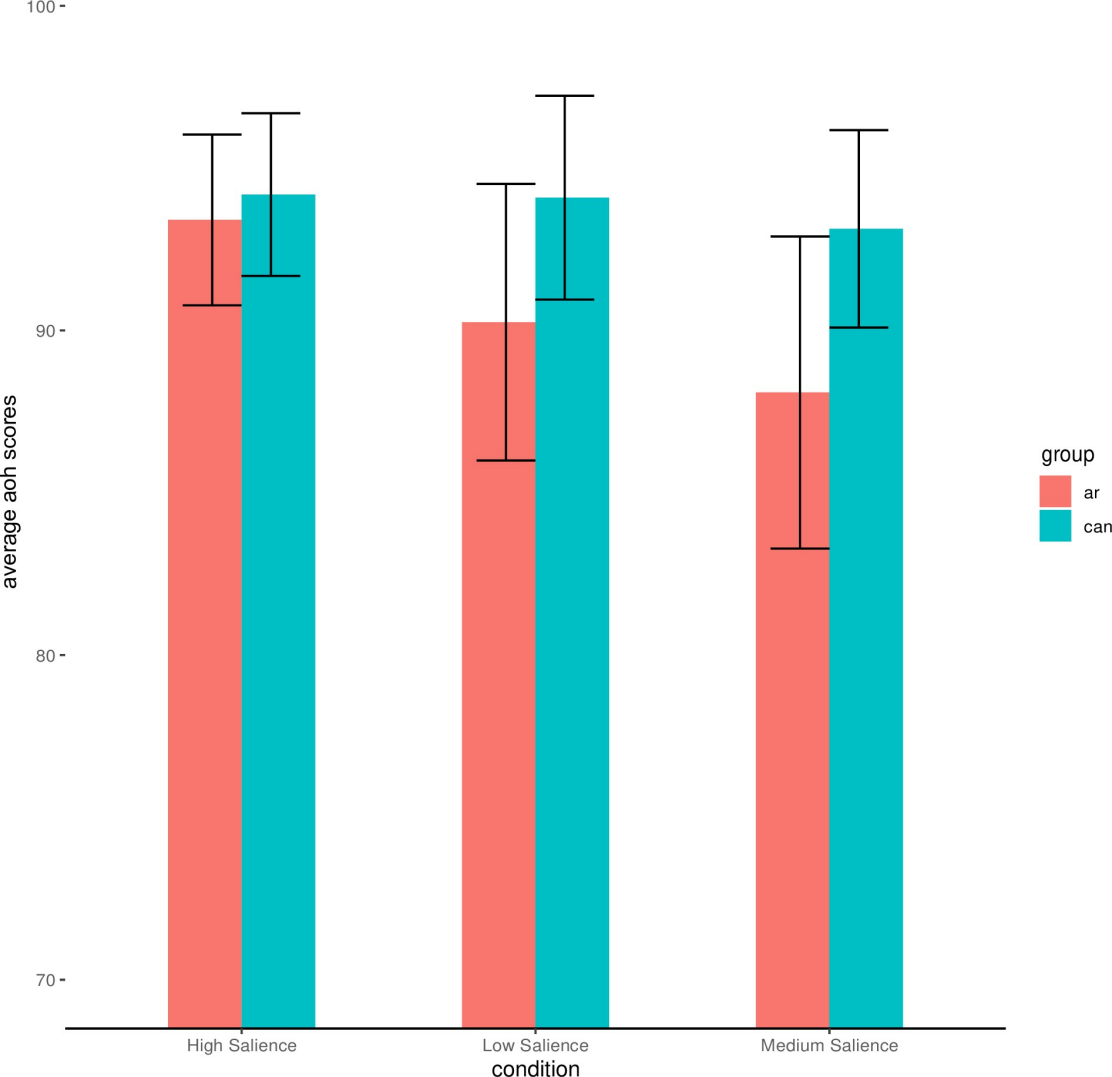
Study 1—Plot of ascent of human ratings for Canadians and Arabs across conditions. Error Bars represent 95% Confidence Intervals.

See Fig 3 for a visualization of these results.

## Study 1 discussion

In this study we presented participants with the AOH scale and manipulated the extent to which an out-group, Arabs, stood out amongst other filler groups. Results from our study indicate that group salience does not have an impact on relative dehumanization, the difference in AOH scores for the out-group minus the in-group. In addition, there was no difference in raw AOH scores for Arabs across the three conditions. These results are in line with previous research examining the methodological aspects of the AOH scale suggesting that it is robust and not subject to change under minor but non-trivial changes to administration protocols. We did see a significant difference in how our Canadian participants rated themselves compared to how they rated Arabs, indicating dehumanization towards Arabs in our sample. As we measured relative dehumanization for our analysis it is possible that the inclusion of Canadian as a label on the AOH scale compromised our low salience condition. In that condition, whilst Arabs was not meant to stand out, Canadians could potentially stand out which may have affected our results. Another possibility is that having “Canadians” indicated in the list of groups cued people to well-known ideas about prejudice and bias, as well as the fact that Canada is known for its successful multiculturalism, thus causing them to provide more socially desirable ratings. We thus decided to run an additional study to further corroborate the apparent lack of a salience effect by running a second study in which the in-group was removed from the AOH scale in all conditions.

## Study 2

### Methods

#### Sample size

As our primary question and analysis plan remains the same all except for questions pertaining to relative dehumanization we elected not to pre-register this study. As such we aimed for the same minimum number of participants, 161, in order to be powered for a moderate effect size (F = .25) for a One-way ANOVA.

#### Participants

We recruited 213 undergraduate students from McMaster University during October of 2022. Similar exclusion criteria from study 1 remained leaving us with a final sample of 176 participants (Mean age = 18.3, SD = 1.7, 81.8% Female).

#### Procedure

Procedure for this study was identical to that of study 1 with the exception that in each salience condition, ‘Canadians’ were removed from all groups.

#### Design and analysis

For study 2 our main analysis was a one-way ANOVA on ascent of human ratings for Arabs.

## Results

Results from the one-way ANOVA revealed a significant main effect of salience condition on ascent ratings for Arabs F(2, 172) = 6.58, p = 0.002; Eta2 = 0.07, 95% CI [0.02, 1.00]. Post-hoc analysis with Bonferroni corrections revealed that ratings for Arabs in the High Salience condition were significantly lower than both the Medium Salience condition (p = .02) and the Low Salience condition (p = .002).

As in study 1 homogeneity of variance as assessed via Barlett’s tests were violated (p < .001) as well as tests for normality of model residuals using Shapiro’s test (p < .001). To address this we computed the non-parametric Kruskal Wallis rank sum test. This revealed a main effect of salience condition on ascent ratings for Arabs H(2) = 11.88, p = < .01. We broke down this effect using the Dunn Test for a non-parametric post-hoc analysis. Results show that ratings in the High Salience condition are lower than in Low Salience condition (p < .01) as well as the Medium Salience condition (p = .02).

**Fig 4:**
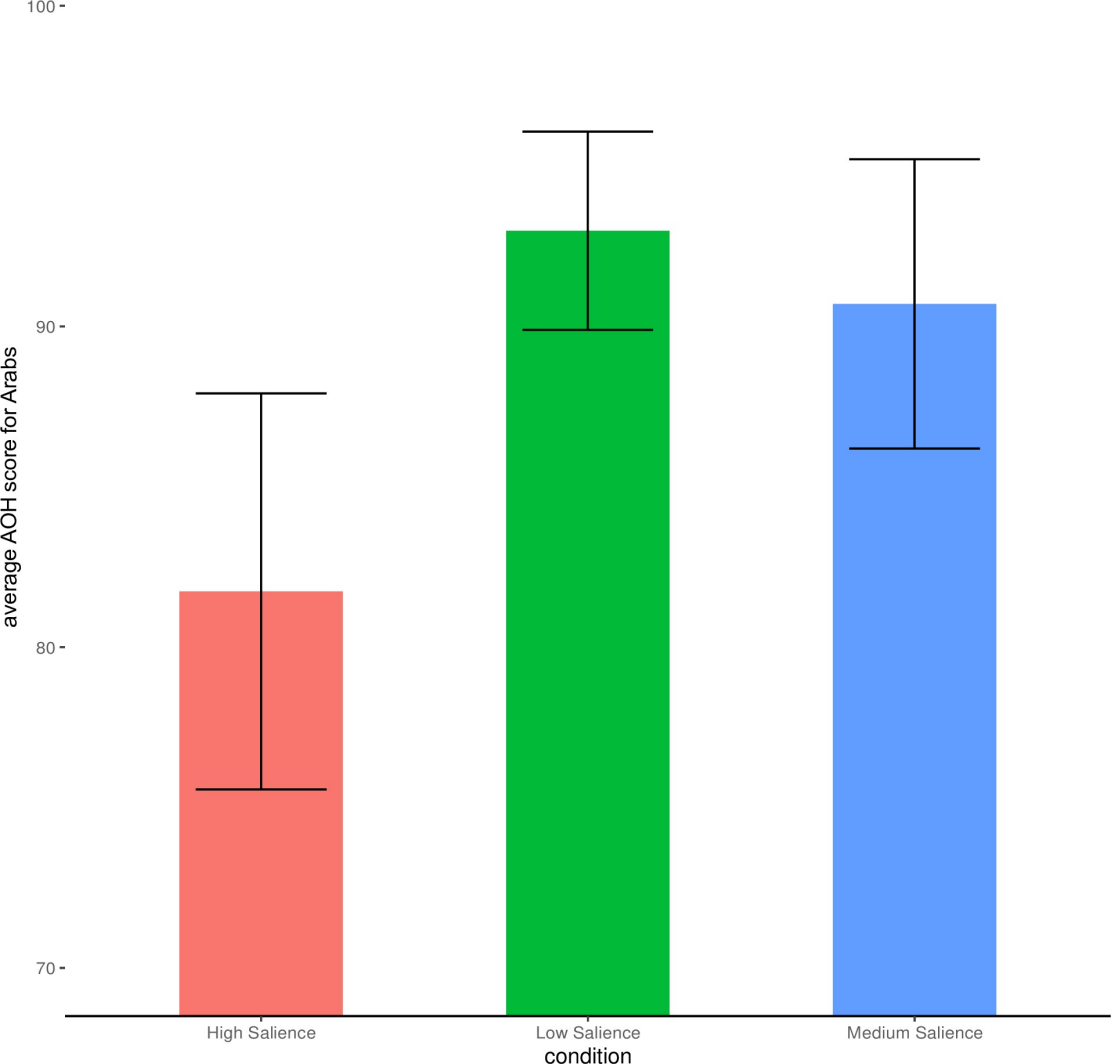
Study 2—Ascent of human ratings for Arabs. Error Bars represent 95% Confidence Intervals.

See Fig 4 for the visualization of the main results from this study.

## Study 2 discussion

Study 2 sought to address concerns over our salience manipulation in study 1 where even in our low salience condition, the in-group of our participants, Canadians, stood out as they are not classified as a marginalized non-western group. We thus conducted a version of our study in which the in-group labels were removed from the AOH scale. Results show that when Arabs stand out amongst other filler groups, participants rate them significantly lower compared to both medium and low salience conditions. It would appear that the incorporation of the in-group of participants on the AOH scale may exert an influence on the manner in which respondents evaluate out-groups in relative terms. Moreover, as an out-group becomes increasingly distinctive among the various groups represented on the AOH scale, there is a tendency for that group to receive lower ratings. Research employing the AOH scale typically incorporates an in-group as a means to calculate relative dehumanization, a pivotal metric in studies investigating this phenomenon [8]. For this reason, the in-group of our participants was included in study 1 in order to assess if ascent dehumanization was present towards our out-group of interest, relative to the in-group.

Results from this study suggest that when the in-group of participants is not included in the scale, some care may be needed in deciding which filler groups are presented, especially when groups will be presented all at once, a common practice when using the scale.

## General discussion

Across two studies we investigated how the choice of filler groups can influence results on the AOH scale. Specifically, we manipulated the salience of a target group of interest, Arabs, in three conditions in which they stood out amongst other filler groups to varying degrees. In study1, we saw no effect of group salience on relative dehumanization, the difference in ratings between the in-group of our participants (Canadians) and Arabs. We also saw no difference in AOH scores for Arabs across each of our salience conditions. This would indicate that filler group choice has no impact on AOH ratings for comparisons of interest. However, in study 2 we omitted the in-group of our participants from the scale. Results found that when Arabs stood out amongst the other filler groups, they were rated significantly lower than in conditions in which they stood out to a moderate degree or did not stand out at all. As such, when participants were not also rating their in-group along with others, salience appears to have impacted how they rate an out-group.

As argued by infrahumanzation theory [2], people will attribute more human qualities, such as uniquely human emotions to their in-group compared to an out-group [1]. It is possible that salience is not as strong of a factor when participants can also rate their in-group on the AOH scale. Perceptually it may be that all other groups stand out as one homogenous other from the in-group. Another possibility is that we may have had 2 high salience conditions in study 1 by having Canadians in our “low salience” condition. There, Canadians would have stood out amongst the other filler groups, as they are not classified as non-western groups subject to prejudice. The decision to include Canadians as a group to be related in study 1 was out of necessity as studies routinely include the in-group of participants on the AOH scale in order to compute measures of relative dehumanization. We also found evidence of ascent dehumanization towards Arabs by our Canadian participants adding to the benefit of its inclusion in our study.

Despite these necessities, we corrected this potential issue in study 2 and found a salience effect on AOH ratings when the in-group label was absent from the scale. Results show that when Arabs stand out amongst filler groups in the high salience condition, they are rated significantly lower compared to the medium and low salience conditions. Our experimental conditions merely placed different groups around our out-group of interest. If participants truly dehumanize this out-group the presence or absence of other groups should not change that sentiment. This may call into question what the AOH scale is tapping into when participants give ratings on it. One possibility is that participants in our study were simply placing Arabs as to where they believe they were in terms of social and political advancement. As prior work has shown it is possible that respondents on the scale have their own understandings of what the AOH scale is meant to gauge, ones that are contrary to what researchers see it measuring [9, 19].

From a methodological standpoint this brings some concerns into how researchers administer the AOH scale. While many studies using the scale do include some in-group / out-group comparison of interest, some do not. Thus, issues of salience should be noted in future studies to address these concerns, especially when researchers are interested in getting ratings for many groups that vary in status. One possible strategy would involve ensuring that filler groups effectively control for social category salience when utilizing filler groups. Nevertheless, further inquiry may be warranted to scrutinize the necessity of incorporating filler groups in research designs, given that select studies have chosen to forgo their inclusion when investigating the phenomenon of ascent dehumanization across distinct groups [24]. Recent work has argued performance on emotion recognition tasks may be improved when filler tasks are included within experiments [34]. Hence, it would be interesting to investigate the potential impact on ratings to target groups in the context of dehumanization research, arising from the presence or absence of filler groups.

To our knowledge this is now the second study to show that changes in the AOH scale’s administration can have an impact on results with [19] showing that dehumanization is lower when participants are forced to select a distinct image on the ascent of man picture as opposed to a slider scale that affords more flexibility. An increasing body of scholarly literature has displayed a heightened emphasis on the methodological aspects associated with the AOH scale. Noteworthy contributions to this endeavor include an examination of the scale’s implementation in controlled laboratory environments, with a particular focus on addressing potential anchoring effects stemming from the initial positioning of the slider scale [18]. In addition, recent research has underscored the versatility of the AOH scale in gauging dehumanization across diverse contextual settings, illustrated by the introduction of a novel reference image featuring insects, as opposed to the customary primates [35]. This contribution significantly augments our ongoing discourse surrounding the scrutiny of methodological considerations related to the adaptation and utilization of the AOH scale, along with its potential implications for research outcomes. As discussed, many studies use the AOH scale in such a manner that groups tend to be presented for ratings all at once. Is salience an issue when groups are presented one at a time? As researchers have attempted to include similar groups to hide the true comparison of interest, results from our study show this approach is useful but may need more refinement. Future research should investigate standardizing a process upon which filler groups, meant to mask researcher comparisons of interest are devised and included.

Alternatively, our results may imply that dehumanization holds a flexible nature, one that can be modulated when other out-groups are present. When other groups of varying status are present, those who stand out the most may be subject to dehumanization especially if those sentiments were already present. Prior research demonstrates that marginalized groups such as women and minorities can be subjected to harsher scrutiny and hostility in workplace environments [36, 37]. If dehumanization can be elicited by the presence of out-groups under certain conditions, could such hostilities be motivated by dehumanization? Results have shown dehumanization and prejudice to be distinct psychological phenomena [38]. However, if identity salience does have an impact on dehumanization, future research should incorporate dehumanization research into issues of inter-group relations especially in workplace scenarios.

Across two studies we examined how out-group salience may impact ratings on the AOH scale when filler groups are used. While more work is needed to tease apart these issues, results show that researchers should take care in how groups are added to the scale when conducting experiments. In addition, research interested in inter-group relations in various contexts may benefit from incorporating psychological work on blatant dehumanization.
